# Enhancement of indirect functional connections with shortest path length in the adult autistic brain

**DOI:** 10.1002/hbm.24777

**Published:** 2019-08-29

**Authors:** Xiaonan Guo, Tiago Simas, Meng‐Chuan Lai, Michael V. Lombardo, Bhismadev Chakrabarti, Amber N. V. Ruigrok, Edward T. Bullmore, Simon Baron‐Cohen, Huafu Chen, John Suckling, MRC AIMS Consortium

**Affiliations:** ^1^ The Clinical Hospital of Chengdu Brain Science Institute, MOE Key Lab for Neuroinformation; School of Life Science and Technology, Center for Information in BioMedicine University of Electronic Science and Technology of China Chengdu People's Republic of China; ^2^ Brain Mapping Unit, Department of Psychiatry University of Cambridge Cambridge UK; ^3^ Centre for Addiction and Mental Health and the Hospital for Sick Children, Department of Psychiatry University of Toronto Toronto Canada; ^4^ Autism Research Centre, Department of Psychiatry University of Cambridge Cambridge UK; ^5^ Department of Psychiatry National Taiwan University Hospital and College of Medicine Taipei Taiwan; ^6^ Laboratory for Autism and Neurodevelopmental Disorders, Center for Neuroscience and Cognitive Systems @UniTn, Italian Institute of Technology Rovereto Italy; ^7^ Centre for Integrative Neuroscience and Neurodynamics, School of Psychology and Clinical Language Sciences University of Reading Reading UK; ^8^ Cambridgeshire and Peterborough NHS Foundation Trust Cambridge UK

**Keywords:** autism, functional connectivity, functional magnetic resonance imaging, resting‐state, semi‐metricity

## Abstract

Autism is a neurodevelopmental condition characterized by atypical brain functional organization. Here we investigated the intrinsic indirect (semi‐metric) connectivity of the functional connectome associated with autism. Resting‐state functional magnetic resonance imaging scans were acquired from 65 neurotypical adults (33 males/32 females) and 61 autistic adults (30 males/31 females). From functional connectivity networks, semi‐metric percentages (SMPs) were calculated to assess the proportion of indirect shortest functional pathways at global, hemisphere, network, and node levels. Group comparisons were then conducted to ascertain differences between autism and neurotypical control groups. Finally, the strength and length of edges were examined to explore the patterns of semi‐metric connections associated with autism. Compared with neurotypical controls, autistic adults displayed significantly higher SMP at all spatial scales, similar to prior observations in adolescents. Differences were primarily in weaker, longer‐distance edges in the majority between networks. However, no significant diagnosis‐by‐sex interaction effects were observed on global SMP. These findings suggest increased indirect functional connectivity in the autistic brain is persistent from adolescence to adulthood and is indicative of reduced functional network integration.

## 1 INTRODUCTION

Autism is a collective term for neurodevelopmental conditions with behavioral difficulties in social reciprocity and social communication, and restricted interests or repetitive behaviors (American Psychiatric Association, 2013). Approximately 1 in 59 children receive an autism diagnosis (Baio et al., 2018), with a reported male‐to‐female ratio around 3:1 (Baio, 2014; Halladay et al., 2015; Lai, Lombardo, Auyeung, Chakrabarti, & Baron‐Cohen, 2015; Loomes, Hull, & Mandy, 2017). Despite extensive genetic and neuroimaging studies, there is currently little consensus on the etiology of autism. A likely reason for the lack of consensus is that the autistic population is heterogeneous at multiple levels of analysis (Lombardo et al., 2018; Lombardo, Lai, & Baron‐Cohen, 2019). Promising ways forward likely need to involve strategies for discovering mechanisms that identify subsets of individuals rather than searching for markers or explanations that apply to the entire autism population.

The advent of functional connectivity derived from BOLD‐sensitive magnetic resonance imaging (MRI) during awake rest has facilitated our understanding of typical functional brain organization as well as differences that are apparent in psychiatric and neurological disorders (Achard, Salvador, Whitcher, Suckling, & Bullmore, 2006; Guo et al., 2016; Li et al., 2016; Woodward & Cascio, 2015). Early theories and functional connectivity studies suggested that local over‐connectivity develops in concert with long‐range under‐connectivity in autism (Belmonte et al., 2004; Just, Cherkassky, Keller, Kana, & Minshew, 2007; Just, Keller, Malave, Kana, & Varma, 2012). However, a caveat to the majority of work in the literature is that observed differences in autism have been made on samples that are predominantly male. Autistic females are generally underrepresented in most research, and thus prior observations are likely to have a male‐bias (Hull, Jacokes, Torgerson, Irimia, & Van Horn, 2017; Lai et al., 2017). Compared to autistic males, our understanding of the brain functional organization in autistic females remains largely unclear.

Complex network analysis has emerged as a powerful way to quantitatively characterize the communication dynamics between functional brain networks (Avena‐Koenigsberger, Misic, & Sporns, 2018; Bullmore & Sporns, 2009; Rubinov & Sporns, 2010). Briefly, the functional connectome can be constructed as graphs that consist of nodes (brain regions) linked by edges representing temporal synchronicity (frequently correlation) between neurophysiological signals. These edges are generally constrained to be sparse by imposing a threshold on the strength of associated synchronicity. The discarding of weaker edges has been undertaken on the assumption that neural communication processes emerge preferentially through strong connections and along shortest paths. However, the shortest path can be calculated only if an overall map of the structure of brain network is known, and it seems improbable that the brain might carry such a map for continuous and instantaneous reference. Information is more likely to be conveyed through the entire, fully connected connectome involving most, if not all edges (Simas & Suckling, 2016; Suckling et al., 2015), especially given temporal variations of brain organization (Allen et al., 2014; Hutchison et al., 2013). The significance of weak links has been widely acknowledged in the information transfer across friendship networks (Granovetter, 1973, 1983); Weaker inter‐personal relationships being the bridges between groups of strongly tied individuals, thus facilitating the sharing of information over long distances. Recognition of the role of weak links in brain networks has recently emerged from upwardly revised estimates of the inter‐areal connection density in mouse and macaque brains, to greater than 60% (Gamanut et al., 2018; Markov et al., 2013; Ypma & Bullmore, 2016). Within these dense networks, weak links are of greater geometric length and evenly spatially distributed across the connectome (Markov et al., 2013; Ypma & Bullmore, 2016).

A semi‐metric edge occurs when the shortest topological path between two regions is a circuitous path involving additional regions rather than the direct path between them. This transitivity violation behavior supports a high degree of redundancy and between‐network interactions in the brain (Simas et al., 2015; Simas & Suckling, 2016). Many real‐world weighted networks have been confirmed to have various degrees of semi‐metric behavior (Rocha, 2002; Tiago & Rocha, 2015). Semi‐metric analyses of brain networks indicate that the functional connectome exhibits high levels of semi‐metricity, and psychiatric disorders are characterized by idiosyncratic semi‐metric patterns (Peeters et al., 2015; Simas et al., 2015; Suckling et al., 2015).

While the core organizational topology of the connectome might be under debate (Bertolero, Yeo, & D'Esposito, 2017; Griffa & Van den Heuvel, 2018), the consensus is that the brain is modular, with strong local connections defining subnetworks that subserve cognitive functions. During early‐life development, a time when atypical development is often first diagnosed, rapid changes occur in connectome that is subject to the competing forces of module segregation for functional specialization, and inter‐module integration to facilitate behaviors combining specializations (Homae et al., 2010). A reduction in this integration appears to be a key characteristic of autism (Abbott et al., 2016; Duan et al., 2017; Keown et al., 2013, 2017), and in particular the switching between local and global processing (Hong et al., 2019). By virtue of their long‐range influence and ubiquity in the connectome, weak links are suggested as a potential substrate for integrative communication, and thus metrics sensitive to their distribution could be informative of neurodevelopmental disorders such as autism. Global network measures of weighted networks, such as efficiency and the size of connected components, are not affected by the removal of weak links (Ypma & Bullmore, 2016). Semi‐metric percentage (SMP) is well positioned to describe the contribution of weak links to the overall network topology, and to be sensitive to their alterations. Indeed, a prior study of semi‐metricity in autism demonstrated overabundant indirect shortest paths in the functional connectome in autistic adolescents (Simas et al., 2015), with the suggestion that network integration via indirect routing increases the dispersion of information flow and possibly also the risk for atypical information processing. Autistic individuals exhibit developmental changes in brain activity and functional connectivity (Guo et al., 2017; Nomi & Uddin, 2015; Uddin, Supekar, & Menon, 2013). Accordingly, a key question is whether semi‐metric differences occur at other developmental stages of autism, such as adulthood.

The current study sought to address several questions: (a) Is there any difference between autistic adults and typical developing (TD) participants in terms of semi‐metric edges of the functional connectome? (b) Does biological sex affect these differences? (c) What is the pattern of differences in semi‐metric edges? (d) Are these altered semi‐metric connections weak or strong, long or short edges? To this end, we explored the semi‐metric behavior of the functional connectome in a sample of sex‐balanced autistic adults (*n* = 61) and TD (*n* = 65) at different spatial scales. We first assessed semi‐metric behavior at the whole‐brain level, then sequentially decomposed the semi‐metric connectome into hemispheres, networks, and nodes to ascertain the pattern of differences in brain regions. In light of the developmental hypothesis of functional connectivity in autism that suggests that autistic adolescents and adults display similar deviation patterns of intrinsic functional connectivity (Uddin et al., 2013), we hypothesized that increased indirect connectivity would be observed in autistic adults.

## 2 MATERIALS AND METHODS

### 2.1 Participants

This analysis included 33 TD males, 32 TD females, 30 autistic males, and 31 autistic females who participated in the UK Medical Research Council Autism Imaging Multicenter Study (MRC AIMS) after providing written informed consent (Table 1). All participants were recruited from the Autism Research Centre, University of Cambridge. The study was approved by the Suffolk Research Ethics Committee, UK. All participants were required to have age ≥ 18 years, be right‐handed and have full‐scale IQ (FIQ) ≥ 70. The inclusion and exclusion criteria for autistic individuals were identical to those of our earlier studies with this dataset (Lai et al., 2010, 2013). Autistic adults received a clinical diagnosis of autistic disorder or Asperger's syndrome according to the criteria in International Classification of Diseases‐10 (World Health Organization, 1992) or Diagnostic and Statistical Manual of Mental Disorders‐IV text revision (American Psychiatric Association, 2000), confirmed by Autism Diagnostic Interview‐Revised (Lord, Rutter, & Le Couteur, 1994). Current symptoms were assessed using the Autism Diagnostic Observation Schedule (ADOS) module 4 (Lord et al., 2000). TD adults were screened and excluded if they have autism either themselves or in their family history. Exclusion criteria for all participants included current or historical psychotic disorders, substance‐use disorders, severe head injury, genetic disorders associated with autism (e.g., fragile X syndrome, tuberous sclerosis), intellectual disability, hyperkinetic disorder, Tourette's disorder, or any other medical condition affecting brain function (e.g., epilepsy). All participants received the assessment of Wechsler Abbreviated Scale of Intelligence measuring intellectual ability (Wechsler, 1999).

**Table 1:** Demographic characteristics of the participants

Mean (SD)	Male TD	Autistic males	Female TD	Autistic females	Statistics^a^	Autism‐TD
	(*n* = 33)	(*n* = 30)	(*n* = 32)	(*n* = 31)		*p*‐value
Sex (m/f)	33/0	30/0	0/32	0/31	‐	.86^b^
Age (years)	28.4 (6.1)	26.9 (7.4)	27.5 (6.3)	28.2 (8.3)	NS	.77
Full‐scale IQ	116.3 (11.6)	112.6 (15.9)	120.7 (8.3)	112.9 (16.5)	MA < FC (*p* = .017) FC > FA (*p* = .023)	Autism < TD (*p* = .021)
Mean FD	0.18 (0.06)	0.27 (0.18)	0.18 (0.08)	0.20 (0.09)	MC < MA (*p* = .015) MA > FC (*p* = .016)	Autism > TD (*p* = .008)
Mean DVARS	1.3 (0.1)	1.3 (0.2)	1.2 (0.2)	1.2 (0.2)	MC > FA (*p* = .011) MA > FC (*p* = .008) MA > FA (*p* = .001)	.65
ADOS^c^						
SC	‐	15.7 (9.4)	‐	9.2 (8.6)	MA > FA (*p* = .002)	‐
RRB	‐	1.0 (1.0)	‐	0.1 (0.3)	MA > FA (*p* < .001)	‐

Abbreviations: ADOS, autism diagnostic observation schedule; FA, autistic females; FC, neurotypical females; FD, framewise displacement; MA, autistic males; MC, neurotypical males; NS, nonsignificant (*p* > .05); RRB, repetitive, restrictive and stereotyped behavior score; SC, social‐communication total score.

a Independent two‐sample *t*‐tests between any two groups, except nonparametric Mann–Whitney tests for ADOS scores (distribution significantly deviant from normal).

b *χ*^2^ test.

c *n* = 30 for autistic males, *n* = 30 for autistic females.

### 2.2 Data acquisition

All MRI data were acquired using a 3 T GE Signa scanner (General Electric Medical Systems, Milwaukee, Wisconsin) at the Cambridge Magnetic Resonance Imaging and Spectroscopy Unit. For the resting‐state functional MRI scan, participants completed a 13 min 39 s scan (625 volumes) with an echo‐planar imaging sequence using the following parameters: repetition time = 1,302 ms; echo time = 30 ms; flip angle = 70°; matrix = 64 × 64; field of view = 240 mm; 22 axial slices; slice thickness = 4 mm; slice gap = 1 mm. The first five volumes of each resting‐state acquisition were discarded to allow for equilibrium of the magnetization leaving 620 volumes for analysis. During acquisition, participants were instructed to rest with eyes closed, but not fall asleep. We also obtained the high‐resolution T1 MRI images utilizing the Driven Equilibrium Single Pulse Observation of T1 (DESPOT1) mapping technique as described in previous studies (Deoni et al., 2008; Ecker et al., 2013; Lai et al., 2013), with the following parameters: 176 contiguous slices; voxel size = 1 × 1 × 1 mm; field of view = 256 mm; repetition time = 1,800 ms; inversion time = 850 ms; flip angle = 20°. These images were used for registration to a standard anatomical atlas.

### 2.3 Data preprocessing

Resting‐state functional MRI data were preprocessed using Analysis of Functional NeuroImages (AFNI, http://afni.nimh.nih.gov/; Cox, 1996) and the Oxford Centre for Functional MRI of the Brain Software Library (FSL, http://fsl.fmrib.ox.ac.uk/fsl/; Smith et al., 2004), according to pipelines that minimize motion artifacts (Patel et al., 2014). Based on previous semi‐metric studies (Peeters et al., 2015; Simas et al., 2015; Suckling et al., 2015), the following preprocessing steps were applied to functional images: slice‐time correction; rigid‐body head motion correction; obliquity transformation to the structural image; affine co‐registration to the skull‐stripped structural image; spatial transformation to the MNI 152 template in Talairach space; spatial smoothing (6 mm full width at half maximum); and a within‐run intensity normalization to a whole‐brain median of 1,000. Processing steps for denoising head motion were then performed including: wavelet despiking using the BrainWavelet Toolbox (http://www.brainwavelet.org/); nuisance signal regression of the six motion parameters and their first order temporal derivatives and ventricular cerebrospinal fluid signal; and high‐pass frequency filtering with a cutoff frequency of 0.02 Hz. The mean framewise displacement (FD) estimated during head motion correction and mean DVARS, the frame‐by‐frame whole‐brain signal change of the denoised preprocessed data, were calculated for each participant (Power, Barnes, Snyder, Schlaggar, & Petersen, 2012). We used a data‐driven wavelet despiking approach to remove head motion confounds (Patel et al., 2014). This method is designed to denoise in the wavelet domain both linear and nonlinear, nonstationary head motion artifacts spanning multiple frequency scales while retaining information from unaffected scales. This approach ensures the temporal continuity of time series without the need for removal of frames, and has been demonstrated to outperform scrubbing and time despiking algorithms (Patel et al., 2014).

### 2.4 Functional connectome construction

The cerebral cortex and cerebellum was segmented into 268 regions of interest (ROIs) using a functional atlas (Shen, Tokoglu, Papademetris, & Constable, 2013). This parcellation scheme optimized time‐course similarity within each brain region yielding a more coherent set of functional subunits, in comparison to an anatomical atlas. Using the same atlas, we also assigned brain regions to eight networks: medial frontal, frontoparietal, default mode, subcortical/cerebellum, motor, Visual I, Visual II, and visual association, following the approach of a previous study (Finn et al., 2015). Under this definition, temporal regions were grouped into different functional networks, such as motor, medial frontal, and frontoparietal networks, according to the functional homogeneity of these brain regions.

We discarded ROIs with incomplete coverage in at least one participant during the MRI scanning (Figure S4), and thus the Visual II network was omitted due to only two regions in this network having full coverage in all participants. As a result, 172 nodes pertaining to seven networks were used (Figure S1). For each participant, the time series over all voxels in each region were averaged to represent the regional time series. Wavelet correlation analysis was applied to construct the connectivity graphs from the regional time series utilizing the maximal overlap discrete wavelet transform (MODWT) to decompose the extracted time series into four frequency bands: Scale 1 (0.192–0.384 Hz), Scale 2 (0.096–0.192 Hz), Scale 3 (0.048–0.096 Hz), and scale 4 (0.024–0.048 Hz; Achard et al., 2006). Scale‐specific inter‐regional functional connectivity was estimated by computing the Pearson's correlation coefficient between wavelet coefficients at each scale. For each individual, a 172‐node, weighted and undirected functional connectivity matrix was derived from the non‐negative correlations at each of the four scales. Negative correlations were excluded from the following analyses by setting them to zero.

### 2.5 SMP analysis

To characterize the semi‐metric behavior of the functional connectome, we first converted the functional connectivity matrix to a distance matrix via the isomorphism:

$$d_{\mathrm{ij}}=\frac{1}{w_{\mathrm{ij}}}-1$$

where *w*_*ij*_ is the functional connectivity and *d*_*ij*_ is the distance between nodes *i* and *j*. The shortest path between any pair of nodes in the distance graph was calculated by Johnson's algorithm (Johnson, 1954). When the shortest path between nodes is the direct edge that joins them, it is defined as a *metric* edge. Conversely, when the shortest path is an indirect edge via additional nodes, it is defined as a *semi‐metric* edge. The SMP, the ratio of the number of semi‐metric edges to the total number of edges from any given set of nodes, was calculated for the whole‐brain connectome as a measure of dispersed communication between regions. This analysis was then performed at the hemisphere, network and node levels by a decomposition of the whole‐brain connectome into subgraphs. The SMP of each node was calculated to assess the semi‐metric behavior associated with that node, as the number of semi‐metric edges in proportion to the total number of edges emanating from that node.

### 2.6 Semi‐metric backbones

A semi‐metric backbone was generated for each group to examine the consistency of semi‐metric edges across participants. The displayed edge on a backbone depicts the percentage of participants within each group that have a semi‐metric edge at that location. Edges where less than 95% of the participants contributed a semi‐metric edge were excluded from the backbone to enhance visualization.

### 2.7 Statistical analyses

We constructed a general linear model to test for SMP differences between autism and TD groups:

$$\mathrm{SMP}=\beta_{0}+\beta_{1}*\text{Diagnosis}+\beta_{2}*\mathrm{Sex}+\beta_{3}*\left(\text{Diagnosis}\times \mathrm{Sex}\right)+\beta_{4}*\mathrm{Age}+\beta_{5}*\mathrm{FIQ}$$

where Diagnosis is autism or TD, Sex is male or female, Diagnosis × Sex is the interaction between diagnosis and sex, Age is the age factor, and FIQ is the covariate for FIQ. In view of the dependency of these statistical tests at different spatial levels, analyses were carried out under a hierarchical scheme. Statistical testing proceeded to the next level of spatial refinement only if the test in the preceding level was significant (*p* < .05). Nonparametric permutation testing (5,000 permutations) was applied to assess the significance of the model coefficients.

We also examined the age effects on global SMP by using a three‐way interaction model:

$$\begin{array}{l} \mathrm{SMP}=\beta_{0}+\beta_{1}*\text{Diagnosis}+\beta_{2}*\mathrm{Sex}+\beta_{3}*\mathrm{Age}+\beta_{4}*\left(\text{Diagnosis}\times \mathrm{Sex}\right)+\beta_{5}*\left(\text{Diagnosis}\times \mathrm{Age}\right)\\ \;+\beta_{6}*\left(\mathrm{Sex}\times \mathrm{Age}\right)+\beta_{7}*\left(\text{Diagnosis}\times \mathrm{Sex}\times \mathrm{Age}\right)+\beta_{8}*\mathrm{FIQ} \end{array}$$

Since no significant age‐related interaction effect was observed, we used the two‐way interaction model in the following analysis for simplification, and age was included as a covariate in the general linear model.

### 2.8 Strength and length of the semi‐metric edges

To identify the strength of links contributing to semi‐metric topology, we derived a set of functional connectomes thresholding the connectivity (i.e., wavelet coefficient correlations) across the range 0–1 with an increment of 0.01. If the connectivity between regions *i* and *j* exceeded a given threshold then this edge was kept in the functional connectome, if not it was set to 0 denoting the absence of direct functional connectivity between the two nodes. We then calculated the SMP for these functional connectomes and an identical general linear model was then applied to explore the main effects of diagnosis on global SMP. A complementary analysis thresholding strong edges was also performed to provide additional support for the relationship between functional connectivity strength and SMP. As previously, nonparametric permutation testing (5,000 permutations) was applied to assess the significance of statistical model.

In view of the dependency between distances and functional connectivity abnormalities observed in previous neuroimaging studies of autism (Belmonte et al., 2004; Just et al., 2007), we examined the average lengths of semi‐metric edges. The anatomical distance between any two regions was defined as the Euclidean distance between their centroids. At a given threshold, R0, the average semi‐metric edge length was calculated for all the semi‐metric edges whose strength r satisfies 0 < *r* < R0. The general linear model was then applied on semi‐metric edge length to explore the main effect of diagnosis. Nonparametric permutation testing (5,000 permutations) was applied to assess the significance of statistical model with the statistical significance set at *p* < .05. To assess the effect of the number of edges, we plotted the distribution of correlation coefficients for each participant, and compared the edge number between autism and TD groups.

Since the removal of edges may remove the connectedness of the connected graph, we calculated the threshold of functional connectivity that separated the connectome into unconnected subgraphs, for each individual. The general linear model and nonparametric permutation testing (5,000 permutations) were again performed to test for group differences. The statistical significance was set at *p* < .05.

### 2.9 Graph theory analysis

To illustrate the sensitivity of SMP in detecting autism‐related differences, we additionally performed conventional network analysis on the whole‐brain, fully connected, weighted functional connectome using the GRETNA package (Wang et al., 2015). Small‐world properties including clustering coefficient, normalized clustering coefficient *γ*, normalized shortest path length *λ*, and small‐worldness *σ*, and network efficiency, including global efficiency and local efficiency were calculated for all non‐negative weighted connections (Latora & Marchiori, 2001; Watts & Strogatz, 1998). The general linear model was then constructed to explore the differences between autism and TD groups on these network measures as previously. Nonparametric permutation testing (5,000 permutations) was applied to assess the significance of statistical model with statistical significance set at *p* < .05.

### 2.10 Correlations between SMP and autism symptoms

The relationships between SMP at the whole‐brain, hemisphere, and network levels and measures of symptom severity in autistic individuals were explored using Spearman correlation coefficients, assuming a monotonic relationship although not necessarily linear. Sex, age, and FIQ were taken as covariates. Autism symptom severity was assessed by social‐communication and repetitive, restrictive and stereotyped behavior scores in ADOS. Bonferroni correction was performed for multiple comparisons with statistical significance set at *p* < .05.

## 3 RESULTS

### 3.1 Demographic and head motion characteristics

There were no significant differences in sex, age, and mean DVARS between autism and TD groups. However, autistic individuals showed lower FIQ and higher mean FD than the TD group. Female TD participants had slightly higher FIQ on average than the autistic males and females. Autistic males had significantly higher ADOS scores than autistic females. Autistic males exhibited greater mean FD than neurotypical males and females. Autistic males showed greater mean DVARS than autistic females and neurotypical females, and autistic females showed smaller mean DVARS than neurotypical males (Table 1).

### 3.2 Semi‐metricity at different frequency scales

**Figure 1:**
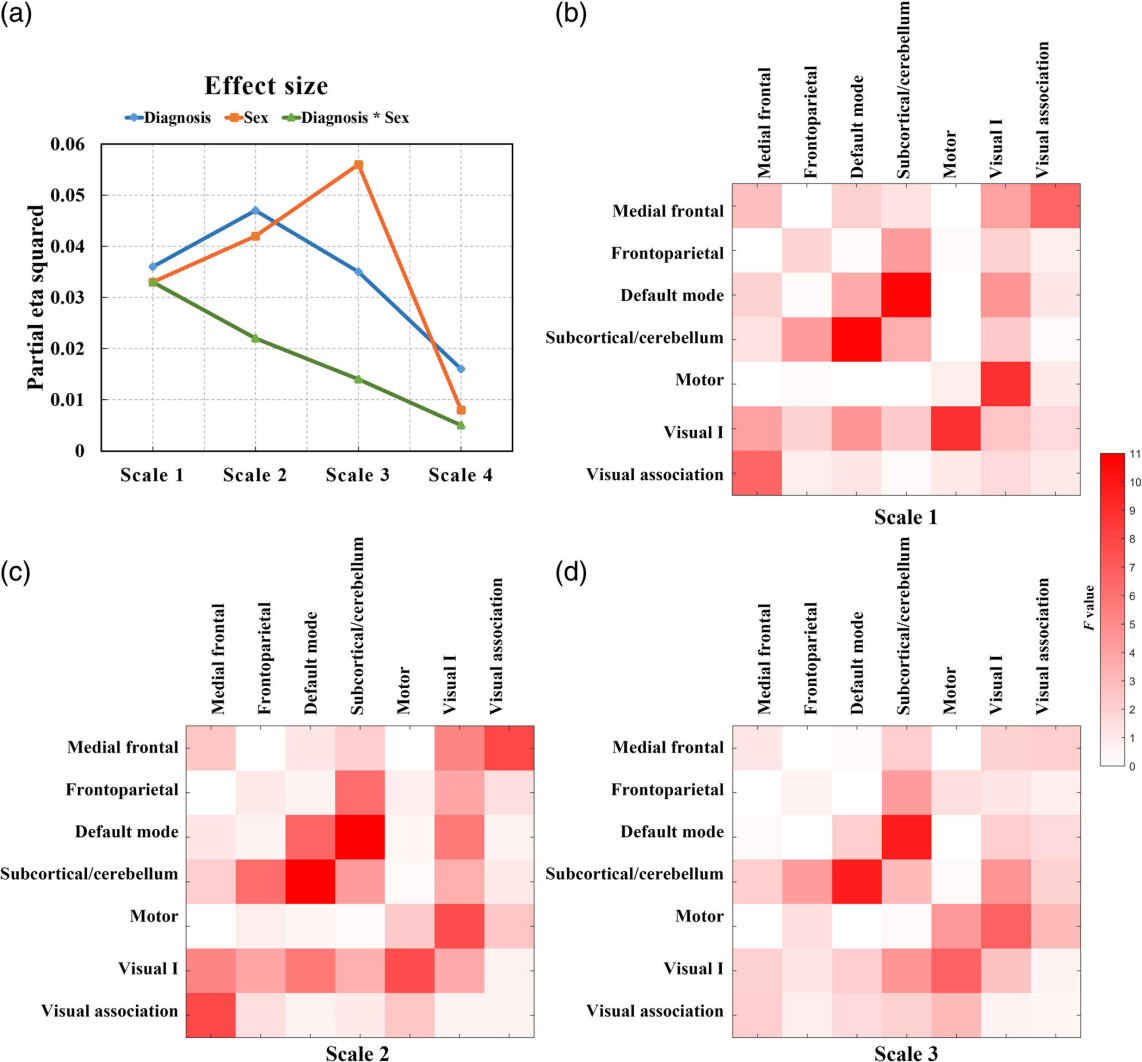
Semi‐metric percentages at different frequency scales. (a) Effect sizes of the main effect of diagnosis, sex, and interaction effect between diagnosis and sex at the whole‐brain level. *F*‐value maps of main effect of diagnosis at network level at scales: (b) 1, (c) 2, and (d) 3 [Color figure can be viewed at http://wileyonlinelibrary.com]

Whole‐brain SMP at scale 4 showed the smallest main effect size of diagnosis, sex and interaction effect between diagnosis and sex (Figure 1). Scale 4 is the lowest frequency band and was close to the cutoff frequency of the filter, and was therefore excluded from our analysis. SMP at scales 1–3 had the same direction of effect and similar effect sizes for the main effect of diagnosis at whole‐brain level, and similar patterns of *F*‐value maps of between‐group difference within and between atlas networks (Figure 1). Since the blood oxygenation level dependent signals in the frequency interval 0.06–0.125 Hz have been demonstrated to detect changes in the brain's functional organization (Bassett, Nelson, Mueller, Camchong, & Lim, 2012; Hermundstad et al., 2013; Suckling et al., 2015), the primary analyses focused on connectivities calculated at scale 3. A factorial analysis of variance results at other scales are provided at Table S1.

### 3.3 Semi‐metric backbones

**Figure 2:**
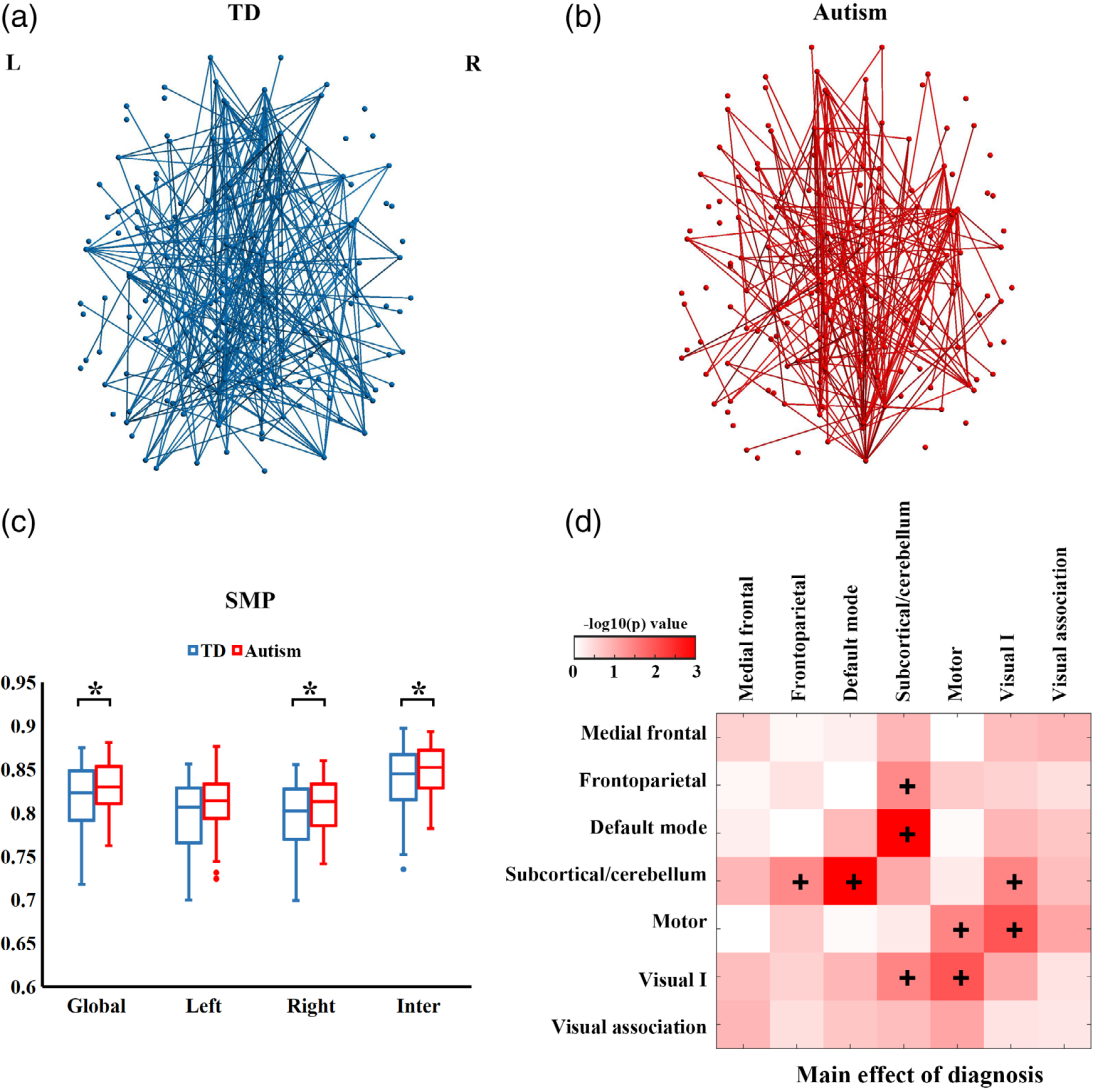
Group differences in semi‐metricity. Axial projections of semi‐metric backbones for (a) TD and (b) autism groups. Edges with the percentage of participants <95% are not shown for clarity. (c) Group comparisons in SMP in the whole‐brain, left, right and inter‐hemisphere edges. The "*" symbol denotes significant group differences at that level (*p* < .05). (d) Main effect of diagnosis at the network level. The + denotes significantly higher SMP in autism compared with TD participants (*p* < .05) [Color figure can be viewed at http://wileyonlinelibrary.com]

Within‐group semi‐metric backbones are shown in Figure 2a,b. Autistic individuals displayed large variations in the percentage of semi‐metric edges compared with the TD group. The number of semi‐metric edges in the backbones for autism and TD groups is 208 and 249, respectively.

### 3.4 SMP differences between autism and TD groups

**Figure 3:**
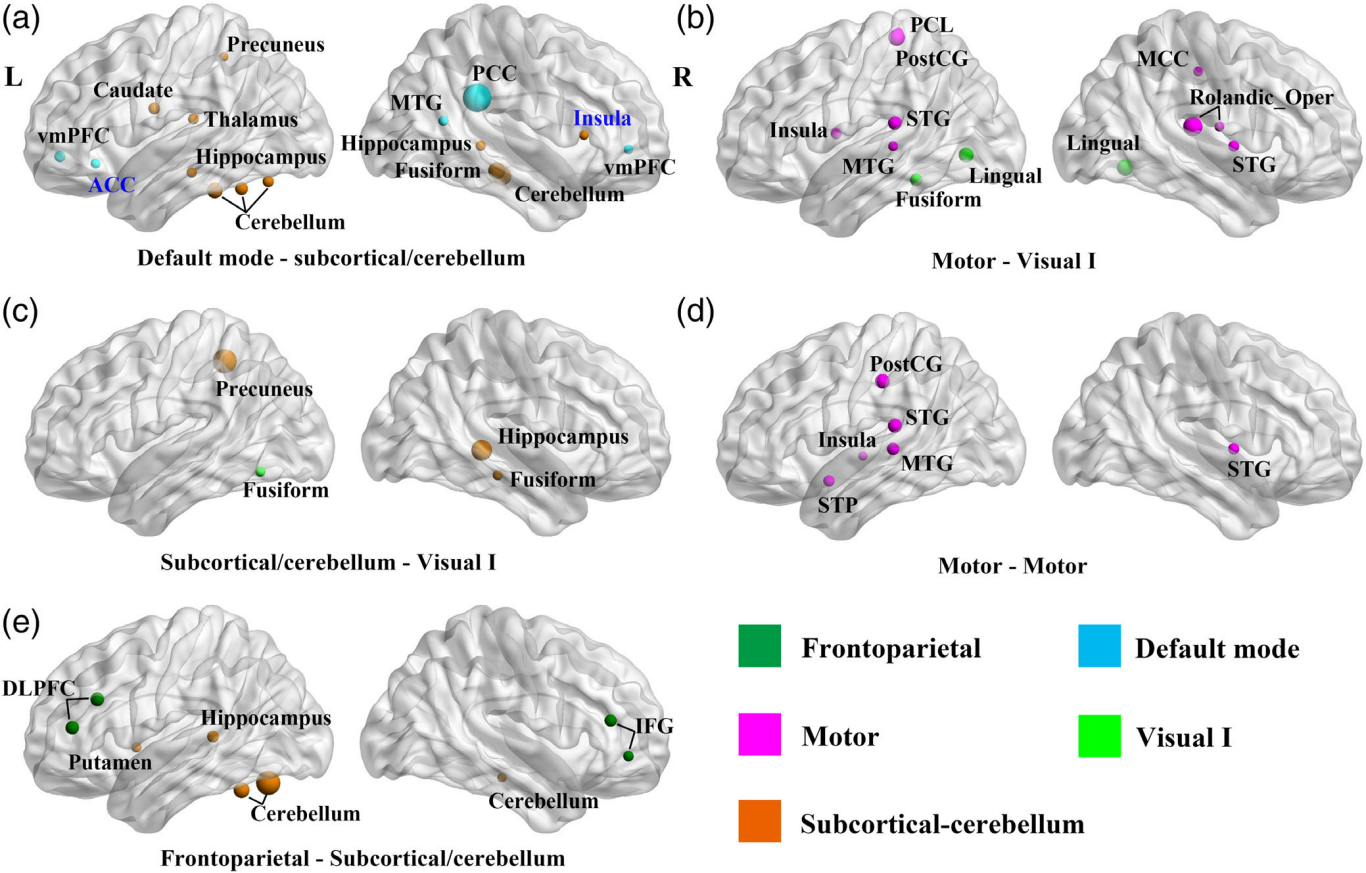
Group differences on node semi‐metric percentage (SMP). Figures are arranged in a descending order according to the effect size of group comparison at the network level, (a) being the largest. The size of the node is proportional to the *F*‐value of the main effect of diagnosis. Only nodes with *p* < .05 are presented. The colors of the nodes represent different networks according to the key. Black node names denote increased SMP in autism, while blue node names denote decreased SMP in autism. ACC, anterior cingulate cortex; DLPFC, dorsal lateral prefrontal cortex; IFG, inferior frontal gyrus; MCC, middle cingulate cortex; MTG, middle temporal gyrus; rolandic_Oper, rolandic operculum; PCC, posterior cingulate cortex; PCL, paracentral lobule; PostCG, postcentral gyrus; STG, superior temporal gyrus; STP, superior temporal pole; vmPFC, ventral medial prefrontal cortex [Color figure can be viewed at http://wileyonlinelibrary.com]

At the whole‐brain level, we observed significant main effects of diagnosis (*F* = 4.35, *p* = .034), sex (*F* = 7.13, *p* = .0076), and age (*F* = 19.55, *p* < .001; Table 2) on SMP. No interaction effect between diagnosis and sex (*F* = 1.67, *p* = .19), or effect of FIQ (*F* = 0.033, *p* = .86) was found. Post‐hoc analyses showed that autistic individuals had significantly increased SMP compared with the TD group (Figure 2c). Analysis at the hemisphere level confirmed that overall, between‐group differences had contributions of higher SMP in the right hemisphere and inter‐hemispheric connections in the autism group than TD group. At the network level, compared with TD group, consistently higher SMP was identified in the autism group in the motor network and between four networks: frontoparietal––subcortical/cerebellum, default mode––subcortical/cerebellum, subcortical/cerebellum––Visual I, motor––Visual I (Figure 2d). Node‐level analysis showed that autistic individuals had higher SMP in multiple brain regions excepting the left anterior cingulate cortex and right insula (Figure 3).

**Table 2:** Factorial analysis of variance on SMP (*F/p* values)

	Global	Left hemisphere	Right hemisphere	Inter‐hemisphere
Main effect of diagnosis	4.35/0.034*	3.73/0.053	4.27/0.035*	3.98/0.042*
Main effect of sex	7.13/0.0076*	5.88/0.017*	10.52/0.001*	5.22/0.022*
Diagnosis * sex	1.67/0.19	1.84/0.174	1.67/0.21	1.30/0.27
Age	19.55/<0.001*	10.13/0.0024*	19.13/<0.001*	22.59/<0.001*
FIQ	0.033/0.86	0.26/0.61	0.17/0.67	0.067/0.80

*F*‐value, the *F* statistic of the *F*‐test on the general linear model.

*p*‐value, the *p* statistic of the nonparametric permutation testing.

* Significant effect of factors (*p* < .05).

### 3.5 SMP differences between males and females

**Figure 4:**
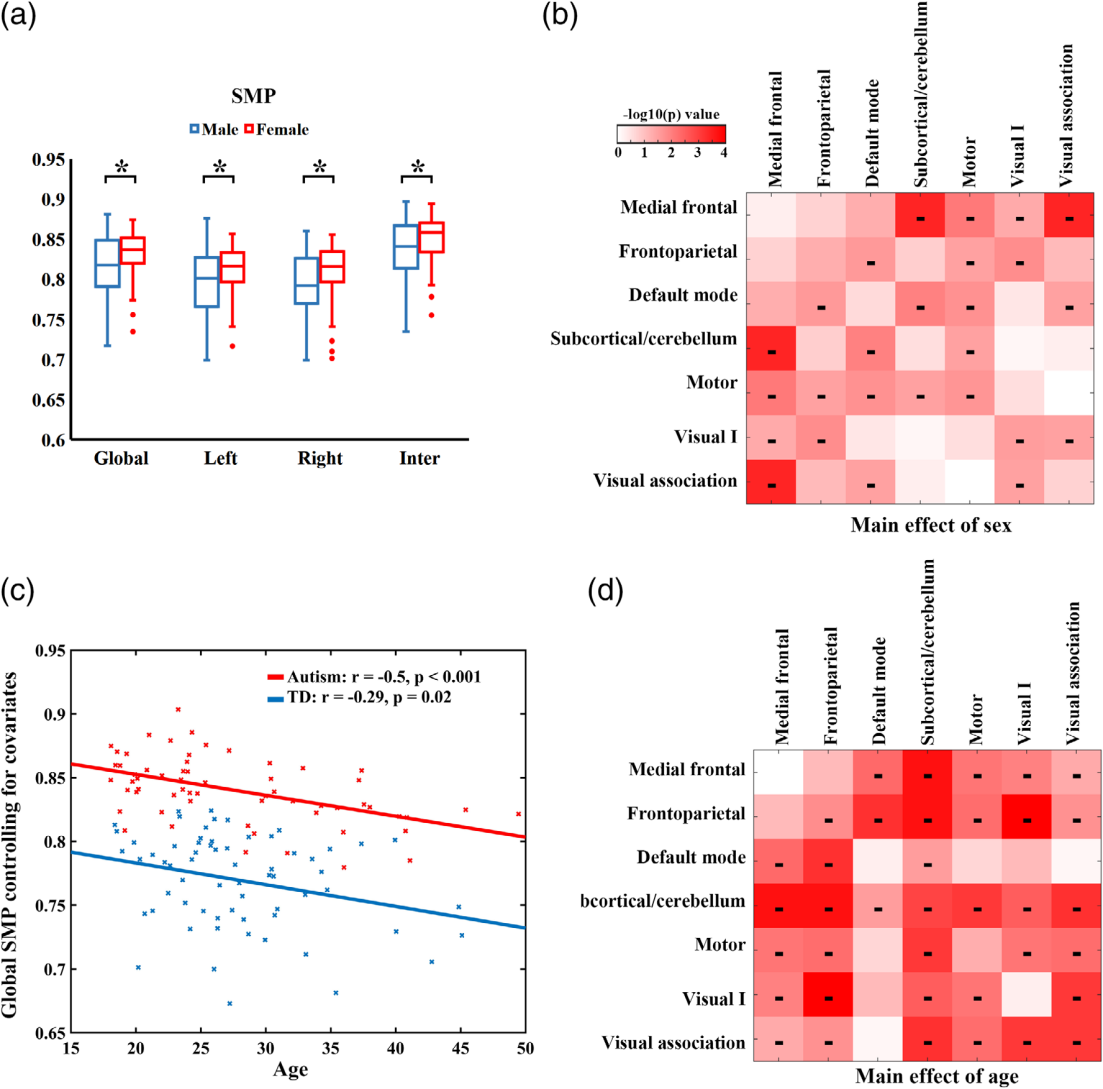
Sex and age effects on SMP. (a) Post‐hoc analyses for main effect of sex on SMP. The "*" symbol denotes significant group differences at that level (*p* < .05). (b) Main effect of sex at the network level. The "‐" symbol denotes significantly lower SMP in males compared with females. (c) The relationship between age and global SMP controlling for sex and FIQ for autism and TD groups. (d) Main effect of age at the network level. The "‐" symbol denotes a significantly negative linear relationship between SMP and age [Color figure can be viewed at http://wileyonlinelibrary.com]

Post‐hoc analysis for the main effect of sex showed that females exhibited higher global SMP than males (Figure 4a). Sex differences on SMP were observed in both hemispheres as well as the inter‐hemispheric edges. Compared with females, males displayed lower SMP in all networks with significant sex differences (Figure 4b).

### 3.6 Age effect on SMP

Post‐hoc analysis for the main effect of age showed that whole‐brain SMP exhibited an age‐dependent reduction. The relationship between age and whole‐brain SMP was plotted for each group controlling for sex and FIQ (Figure 4c). All the hemispheric divisions showed negative correlations between age and SMP (Figure S2). Examination of age effects revealed age‐related decreases in SMP in a large proportion of networks (Figure 4d). A model with a three‐way interaction did not find any significant interplay between diagnosis and age (*F* = 0.0037, *p* = .95), between sex and age (*F* = 0.13, *p* = .71), or among diagnosis, sex and age (*F* = 2.40, *p* = .13) on global SMP.

### 3.7 Strength and length of semi‐metric edges

**Figure 5:**
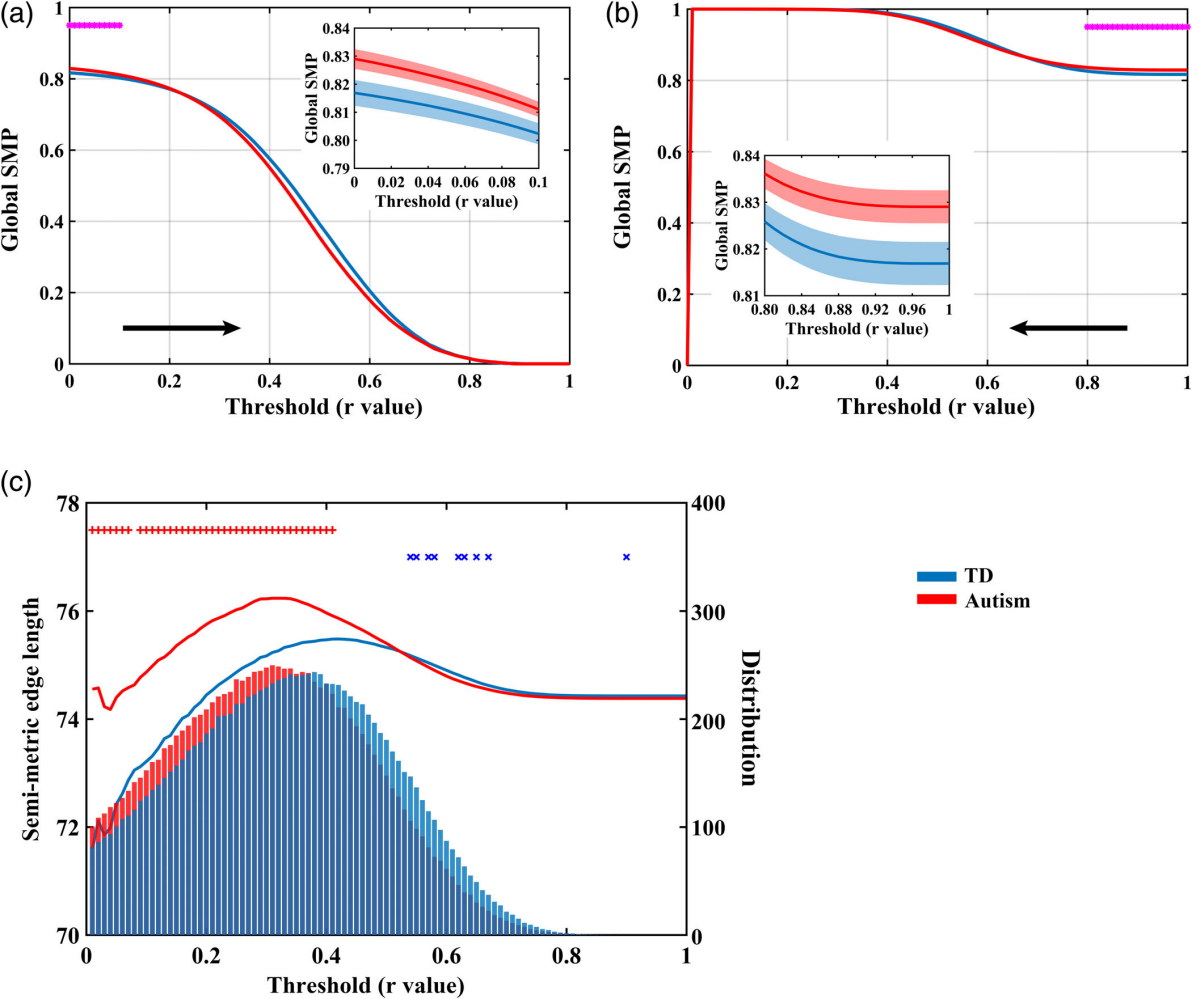
Strength and length of semi‐metric edges. Main effect of diagnosis on global semi‐metric percentages (SMP) when removing (a) low strength edges and (b) high strength edges. The "*" symbol denotes significant group differences (*p* < .05). Detailed information on significant group differences are plotted in the mini‐panel. Shaded regions represent standard errors. (c) Average semi‐metric edge length and the distribution of semi‐metric edges. Line plots denote the relationship between correlation coefficients and semi‐metric edge lengths, and the histogram denotes the average number of semi‐metric edges within each group. The + denotes significant main effects of diagnosis on semi‐metric edge length (*p* < .05; red: autism > TD; blue: autism < TD); the "×" symbol denotes significant main effects of diagnosis on the number of semi‐metric edges (*p* < .05; red: autism > TD; blue: autism < TD) [Color figure can be viewed at http://wileyonlinelibrary.com]

Compared with the TD group, autistic individuals showed significantly higher SMP when removing low strength edges from the connectome in the threshold range 0–0.1 (Figure 5a). In both groups, SMP declined as the threshold increased. The complementary analysis removing high strength edges showed significantly higher SMP in autism than TD groups in the threshold range 0.8–1 (Figure 5b). Group comparisons on semi‐metric edge lengths showed that autistic individuals had on average longer semi‐metric edges than the TD group (Figure 5c). Edge length distribution for all edges and metric edges are provided in Figure S3. No significant main effect of diagnosis (*F* = 0.67, *p* = .42), main effect of sex (*F* = 0.65, *p* = .42), or interaction effect between diagnosis and sex (*F* = 0.31, *p* = .59) was observed on the threshold of functional connectivity graph that first separated the connectome into unconnected subgraphs.

### 3.8 Graph theory analysis

Conventional graph theory analysis of whole‐brain unthresholded functional connectomes showed no significant differences between autism and TD groups in clustering coefficient (*F* = 3.79, *p* = .058), normalized clustering coefficient *γ* (*F* = 1.44, *p* = .24), normalized shortest path length *λ* (*F* = 2.54, *p* = .12), small‐worldness *σ* (*F* = 3.69, *p* = .058), global efficiency (*F* = 3.58, *p* = .06), and local efficiency (*F* = 3.44, *p* = .07). No significant interaction effect between diagnosis and sex was observed in clustering coefficient (*F* = 2.04, *p* = .15), normalized clustering coefficient *γ* (*F* = 1.33, *p* = .26), normalized shortest path length *λ* (*F* = 2.10, *p* = .15), small‐worldness *σ* (*F* = 2.83, *p* = .09), global efficiency (*F* = 1.65, *p* = .20), and local efficiency (*F* = 1.54, *p* = .21).

### 3.9 Correlations with autism symptoms

No significant correlations were found between whole‐brain SMP and ADOS scores in autistic individuals.

## 4 DISCUSSION

This study examined indirect functional connectivity during the resting‐state in autistic adults and typically developing participants. Compared with the TD group, prominent increases in SMP were revealed in autistic adults at the whole‐brain, hemisphere, network, and node levels (Figures 2 and 3). Notably, strength and length analyses on semi‐metric functional connectivity indicated that weaker edges contributed preferentially to semi‐metric topology (Figure 5), with semi‐metric functional connectivity associated with longer edges on average in autistic individuals. However, in this sex‐balanced sample we did not find significant diagnosis‐by‐sex interaction effects, indicating that the increases in SMP in autism compared to TD participants are not dependent on sex, or not detectable with the current sample size. Nevertheless, conventional graph theory analyses on the whole‐brain functional connectome failed to reveal autism‐related alterations in network properties, while semi‐metric analyses detected topological changes specific to autism, confirming the sensitivity of semi‐metricity when weak links are included in the connectome.

Autism is a neurodevelopmental condition that has been characterized by abnormalities in intrinsic functional connectivity (Belmonte et al., 2004). In autism, the primary contribution to positive deviations in whole‐brain semi‐metricity came from higher SMP in the intra/internetworks including the frontoparietal, default mode, subcortical/cerebellum, motor and Visual I networks. These topological findings using semi‐metricity extend previous autism‐related observations showing changes in functional connectivity involving the default mode network (Guo et al., 2019; Lynch et al., 2013), subcortical‐cortical networks (Cerliani et al., 2015), cerebro‐cerebellar networks (Khan et al., 2015) and visual‐motor networks (Nebel et al., 2016). The increases in semi‐metricity in autism were consistently observed across different frequency scales (Scales 1–3), and even in high‐frequency bands (Figure 1). Emerging evidence has demonstrated the persistence of resting‐state spontaneous fluctuations above 0.1 Hz (Chen & Glover, 2015; Peeters et al., 2015; Yuan, Wang, Zang, & Liu, 2014), and these findings collectively highlight the importance of characterizing high‐frequency brain activities in this and future studies of developmental disorders. Although semi‐metric edges were largely increased in number, semi‐metric backbones of autism showed more variation (i.e., a reduced number of edges represented) in their spatial distribution than that of the TD group. This inconsistency in location of the shortest indirect functional connections is in agreement with the evidence of a general increase in heterogeneity of imaging measures in autism (Chen et al., 2018; Jeste & Geschwind, 2014).

The higher SMP across a wide range of spatial scales that was observed in autistic adults closely coincides with previous findings in autistic adolescents (Simas et al., 2015). Although not a longitudinally designed experiment, age‐related changes in semi‐metricity in autism were strong, but did not differ in their rate from TD participants (Figure 4c). The developmental model of functional connectivity in autism proposes the presence of hyper‐connectivity in autistic children with hypo‐connectivity emerging in adolescence and adulthood (Uddin et al., 2013). In contrast to these putative changes in connectivity strength, typically among only the strongest connections, differences in semi‐metricity relative to TD individuals, which are dependent on the weaker connections, appear stable and persistent between the second to fifth decades of life. How early in life these changes occur is unknown, but tracking this measure in young children could give insights into the neurobiological etiology of autism.

While sex differences are well established for measures of resting‐state functional connectivity in TD individuals (Biswal et al., 2010; Ritchie et al., 2018), these differences are currently poorly understood in autism partly due to the limited number of autistic females participating in neuroimaging studies. The question arises whether the brain functional organization differs between autistic males and females after taking typical sex differences into account. To the best of our knowledge, only three studies have assessed resting‐state functional connectivity in autistic males and females (Alaerts, Swinnen, & Wenderoth, 2016; Di & Biswal, 2016; Kozhemiako et al., 2019). Largely sex‐independent functional connectivity differences were observed in autism, with sex‐dependent differences only identified between the precuneus and medial cerebellum/dorsal frontal cortex (Di & Biswal, 2016). Using seed‐based and whole‐brain functional connectivity analyses, Alaerts et al. (2016) found that autistic males predominantly displayed hypo‐connectivity while autistic females predominantly exhibited hyper‐connectivity compared to sex‐matched typical controls. Interhemispheric homotopic functional connectivity was showed to follow different development trajectories between autistic males and females (Kozhemiako et al., 2019). In the current study, failure to detect a diagnosis‐by‐sex interaction effect on global SMP (Table 2) suggests sex‐independent semi‐metric changes in autistic adults, although small sample sizes reduce statistical power. Alternatively, there may be interactions in localized regions that are undetectable at the whole‐brain level, and our hierarchical approach to statistical testing traded sensitivity for robustness. There was a weak, but significant interaction at the highest frequencies (Table S2), which may be worthy of future attention, but on current evidence global increases in SMP do not discriminate between autistic males and females. Future studies with a larger sample size may allow us to better examine the role of sex in heterogeneity, focusing on consensus regions of sexual differentiation in brain structure or function while bearing in mind that the characteristic patterns of autism often differ in spatial distribution in men and women (Alaerts et al., 2016; Lai et al., 2013).

Global changes in semi‐metricity were not correlated to overall symptom scores, although there is no particular reason to a priori hypothesize this relationship and again, our hierarchical approach to statistical testing might have been compromised. Increases in the number of circuitous shortest functional pathways in autism result in the involvement of additional nodes in brain communications, and this may have a bearing on specific cognitive and behavioral styles associated with autism. The SMP only implies the origins and destinations between which pathways of preferential information flow might occur, in particular between the default mode network, visual and motor systems, frontal‐parietal axis, and sub‐cortical/cerebellum (Figures 2 and 3). Mapping the routes through the brain that form the set of shortest pathways and considering their variation across individuals in close detail (Leming, Su, Chattopadhyay, & Suckling, 2019) may give clues to the networks implicated and allow the generation of hypotheses connecting semi‐metricity and cognitive and behavioral styles.

Previous graph theoretical studies have suggested that brain network organization in autistic adolescents and adults exhibits less clustering, reduced local efficiency and higher global efficiency over a certain range of thresholds (Itahashi et al., 2014; Rudie, Brown, et al., 2012). Such findings have been associated with enhanced randomness of the functional connectome (Rudie, Brown, et al., 2012). In general, while there is a consensus that there are significant differences in connectivity associated with autism, the extant literature is somewhat conflicted with regards to the distribution of the effects, with a meta‐analysis of functional MRI connectivity indicating local under‐connectivity (Lau, Leung, & Lau, 2019) while that for EEG and MEG studies indicates mixed local over‐connectivity and under‐connectivity dependent of frequency (O'Reilly, Lewis, & Elsabbagh, 2017), and the absence of converging evidence remains (Hull et al., 2017). However, most if not all prior functional connectivity studies with MRI imposed thresholds on edge strengths that discarded weaker edges; the inclusion of weak edges does not strongly impact on graph theoretical metrics sensitive to weighted shortest path lengths, such as efficiency (Ypma & Bullmore, 2016). This study, and other emerging evidence, demonstrates the important role that weak links have, particularly in the long‐range integration of the modular organization of the brain, to which semi‐metric analysis appears sensitive in a consistent manner across age groups.

Analysis of the strength of semi‐metric edges confirmed the role weaker edges play in the increased number of indirect shortest paths in autism. Furthermore, changes to semi‐metric topology in autism are focused on longer distance weaker edges compared to the TD group (Figure 5). Functional connectivity studies have previously reported distance‐dependent patterns of differences in autism (Courchesne & Pierce, 2005; Just et al., 2007; Long, Duan, Mantini, & Chen, 2016). Belmonte et al. (2004) proposed that autistic individuals may exhibit widespread, reduced long‐distance functional coordination and increased local functional connectivity among brain regions, although this has not been universally replicated (Supekar et al., 2013). Our findings support the effect of anatomical distance on network changes in autism from the perspective of semi‐metric topology, and thus support the argument that anatomical distance is an important consideration in future functional connectivity studies of autism (Long et al., 2016).

Increased semi‐metricity is suggestive of greater dispersal of communication across the brain in autistic individuals; that is, autistic individuals synchronously co‐activated an increased number of brain regions during wakeful rest. In the current study, significant contributions to the global increases in SMP were primarily localized between networks, highlighting atypical, large‐scale internetwork coordination in autism between visual and default mode networks as well as subcortical regions known to the involved in social cognition (Abbott et al., 2016; Duan et al., 2017; Hagen, Stoyanova, Baron‐Cohen, & Calder, 2012). The interaction of these networks is proposed as the mechanism for the top‐down control of behavior (Posner & Petersen, 1990), and the involvement of additional nodes in between‐network communication may be a source of the reduced integration that is a key feature of connectivity in autism (Hull et al., 2017).

Typical neurodevelopment is accompanied by increased functional integration and segregation of large‐scale brain networks (Fair et al., 2007; Fair et al., 2008; Stevens, Pearlson, & Calhoun, 2009). However, a growing number of studies have reported reduced functional integration and segregation of brain networks in autistic individuals (Fishman, Datko, Cabrera, Carper, & Muller, 2015; Keown et al., 2017; Rudie, Shehzad, et al., 2012). Invoking Granovetter's idea of weak links as the connections between strongly connected peer groups, the introduction of intermediaries in those connections could dilute or interfere with message passing and weaken their coordinated function. This atypical communication may appear over a limited period of time in early life as a divergence in functional brain organization, possibly associated with altered neural development (Courchesne et al., 2007), that then persists across the life span. Our findings with semi‐metricity provide additional support for future studies exploring the importance of brain network development in understanding the cognitive and behavioral styles of autistic individuals.

## 5 LIMITATIONS

Several limitations of the current study should be noted. First, having obtained similar effects in semi‐metricity in both autistic adolescents and adults (Simas et al., 2015), we have conjectured developmental origins of changes, but using a cross‐sectional design. Future longitudinal studies are needed to delineate the neurodevelopmental trajectory of semi‐metric topology in autism, especially during early stages of life. Second, the Visual II network was excluded from the current study due to incomplete coverage during MRI scanning, giving rise to the unintended implication of the absence of an effect in this network. Third, it remains unclear how semi‐metric topology interacts with cognition and physiological processes. Semi‐metric behavior of the functional connectome is assumed to reveal functional relevance of indirect paths. Such circuitous functional connections suggest a dispersion of information flow among and between brain networks (Simas et al., 2015; Simas & Suckling, 2016). Exploring semi‐metricity during cognition tasks may facilitate better characterization of network integration in autism, as would focusing on behavior that is subserved by the networks. Fourth, larger sample sizes are needed for future studies, to better examine the role of heterogeneity by sex. Although the current study has a fair number of females compared to other work, it still represents a relatively small sample. Statistical power could increase substantially with more data, and may allow the discovery of effects that interact with sex.

## 6 CONCLUSION

This study extends our understanding of semi‐metric topology to autistic adult individuals who, like adolescent individuals, have increased circuitous shortest functional pathways at whole‐brain, hemisphere, network, and node levels. Moreover, the older age of the autistic participants compared to those in our previous study (Simas et al., 2015), and the absence of any difference in the rate of change of semi‐metricity with age, suggest a biomarker that is persistent from adolescence to adulthood. Changes to semi‐metricity in autism are attributed to weaker and longer‐distance functional connectivity and reduced integration of functional networks, which aligns and encompasses much of the extant literature. Encouraged by these results, further replications in larger datasets and detailed mapping of functional shortest pathways offer a consistent and informative approach to the complex alterations in functional architecture associated with autism.

## DISCLOSURES

E.T.B. is employed half‐time by the University of Cambridge and half‐time at GlaxoSmithKline plc (GSK); he holds stock in GSK. All other authors have no conflict of interests to declare.

## Supporting information


**Data S1** Table S1. Factorial analysis of variance in SMP at other frequency scales.
**Figure S1.** Node and network definitions.
**Figure S2. Relationships between age and SMP at the hemisphere level for all participants.** The effects of diagnosis, sex, diagnosis‐by‐sex and FIQ were regressed out from the model.
**Figure S3. Edge length and distribution of all edges (A) and metric edges (B).** Line plot denotes the relationship between correlation coefficients and edge length, and histogram denotes the average number of edges within‐group. +, significant main effect of diagnosis on edge length (*p* < .05; red: autism > TD; blue: autism < TD); ×, significant main effect of diagnosis on number of edges (*p* < .05; red: autism > TD; blue: autism < TD).
**Figure S4. Regions with incomplete coverage across participants.** Each of eight networks has several regions, primarily located in the parietal lobe and cerebellum, that did not have full coverage in all participants.

Data S2: Supporting Information

## Data Availability

The data that support the findings of this study are available in Supporting Information S2.
