# Supplementary material for: Enhancement of indirect functional connections with shortest path length in the adult autistic brain
Source: Hum Brain Mapp. 2019 Aug 29;40(18):5354–69. doi: 10.1002/hbm.24777 (PMC6864892; doi:10.1002/hbm.24777)
Supplement: Supplementary file 1 — Data S1 Table S1. Factorial analysis of variance in SMP at other frequency scales. Figure S1. Node and network definitions. Figure S2. Relationships between age and SMP at the hemisphere level for all participants. The effects of diagnosis, sex, diagnosis‐by‐sex and FIQ were regressed out from the model. Figure S3. Edge length and distribution of all edges (A) and metric edges (B). Line plot denotes the relationship between correlation coefficients and edge length, and histogram denotes the average number of edges within‐group. +, significant main effect of diagnosis on edge length (p < .05; red: autism > TD; blue: autism < TD); ×, significant main effect of diagnosis on number of edges (p < .05; red: autism > TD; blue: autism < TD). Figure S4. Regions with incomplete coverage across participants. Each of eight networks has several regions, primarily located in the parietal lobe and cerebellum, that did not have full coverage in all participants. [file HBM-40-5354-s001.docx]

**Supplementary materials 1**


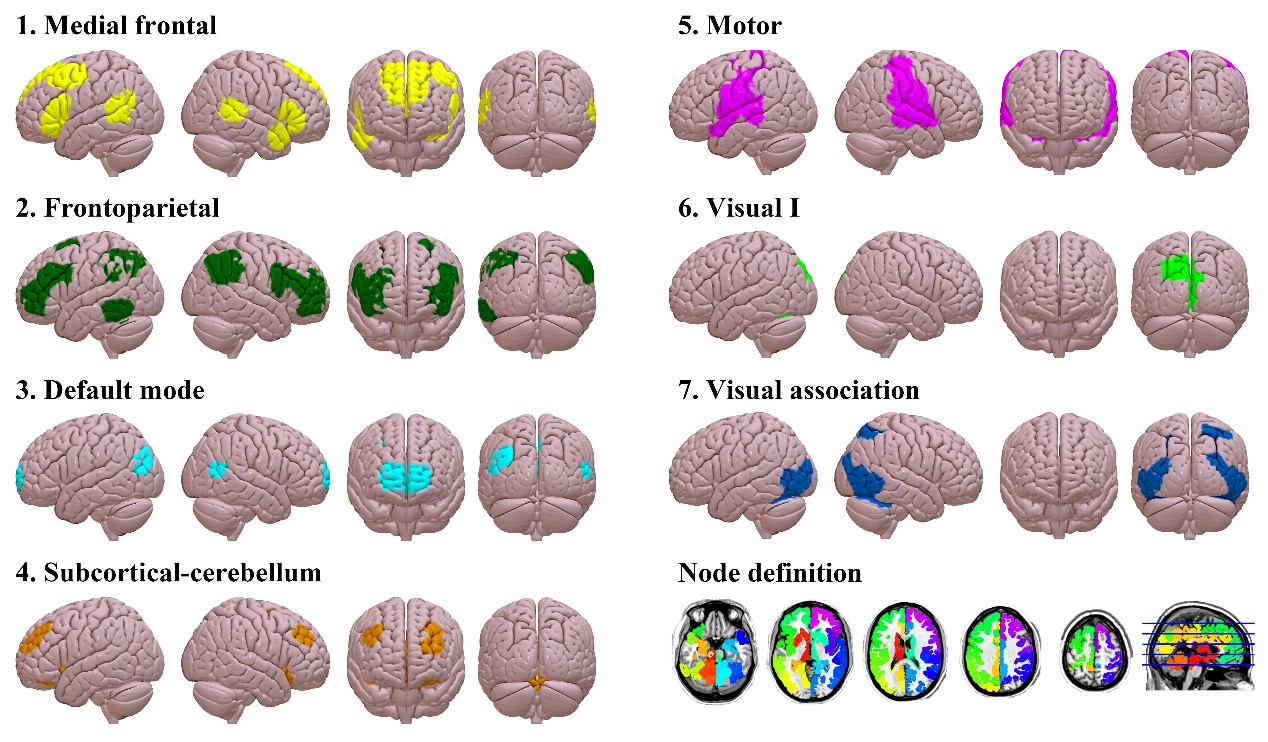


**Figure S1. Node and network definitions.**

**Table S1. Factorial analysis of variance in SMP at other frequency scales.**

| *P* value | Global | Left hemisphere | Right hemisphere | Inter-hemisphere |
| --- | --- | --- | --- | --- |
| Scale 1 (0.19 - 0.38 Hz) | | | | |
| Main effect of diagnosis | 0.035* | 0.08 | 0.065 | 0.021* |
| Main effect of sex | 0.047* | 0.05 | 0.018* | 0.11 |
| Diagnosis * sex | 0.045* | 0.0058* | 0.28 | 0.08 |
| Age | <0.001* | <0.001* | <0.001* | <0.001* |
| FIQ | 0.92 | 0.89 | 0.37 | 0.71 |
| Scale 2 (0.096 – 0.19 Hz) | | | | |
| Main effect of diagnosis | 0.014* | 0.032* | 0.018* | 0.012* |
| Main effect of sex | 0.022* | 0.030* | 0.0076* | 0.051 |
| Diagnosis * sex | 0.11 | 0.027* | 0.30 | 0.15 |
| Age | <0.001* | <0.001* | <0.001* | <0.001* |
| FIQ | 0.87 | 0.87 | 0.37 | 0.79 |

*P* value, the *p* statistic of the non-parametric permutation testing.

*, significant effect of factors (*p* < 0.05).


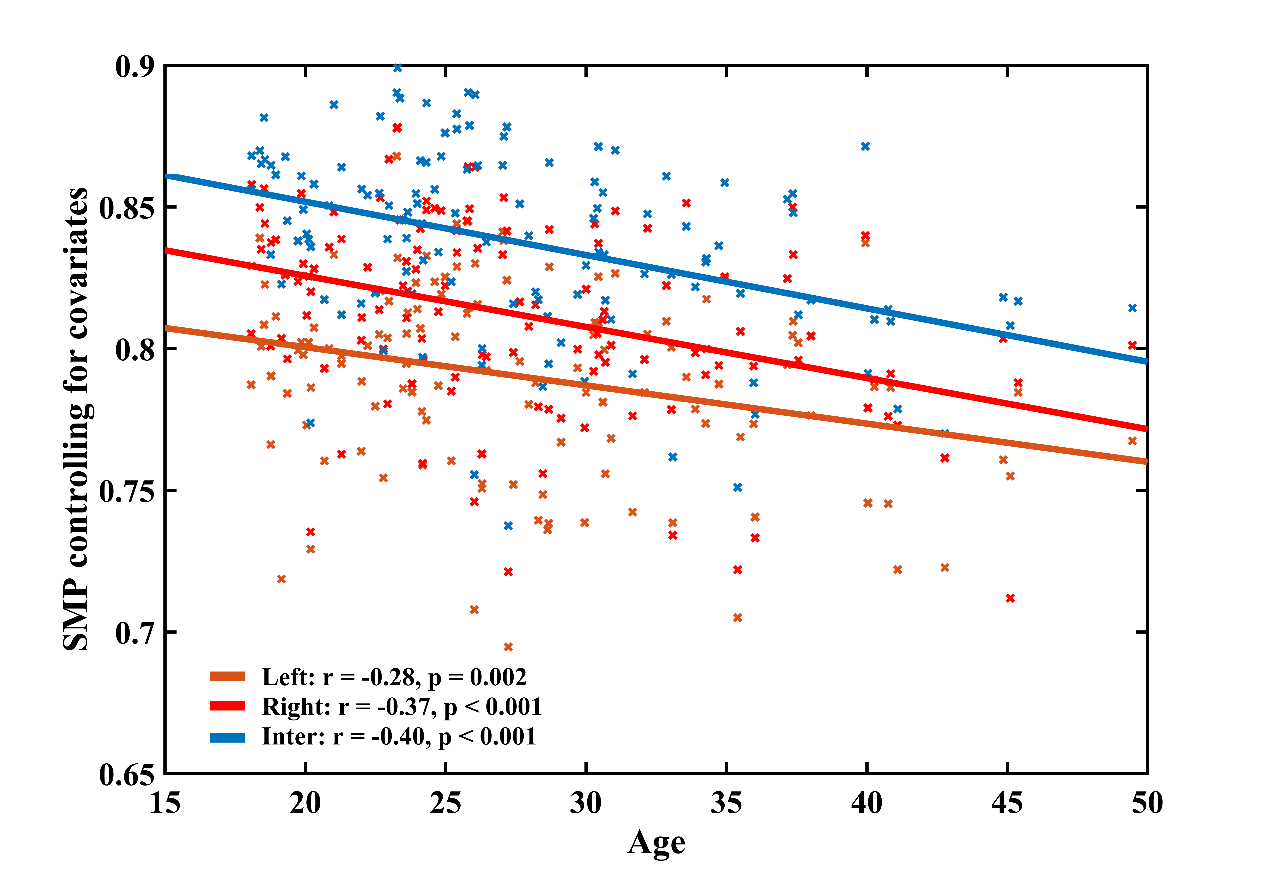


**Figure S2. Relationships between age and SMP at the hemisphere level for all participants.** The effects of diagnosis, sex, diagnosis-by-sex and FIQ were regressed out from the model.


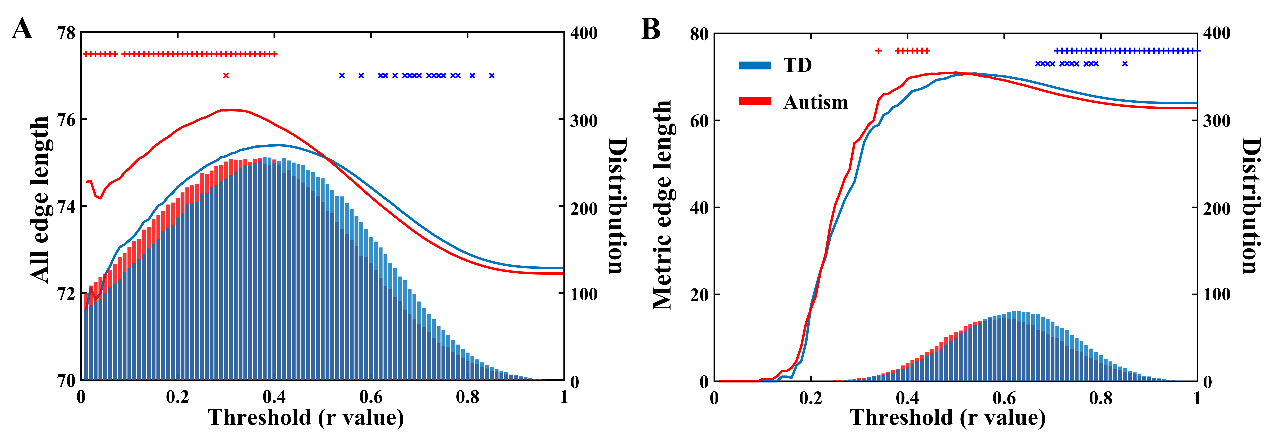


**Figure S3. Edge length and distribution of all edges (A) and metric edges (B).** Line plot denotes the relationship between correlation coefficients and edge length, and histogram denotes the average number of edges within group. +, significant main effect of diagnosis on edge length (*p* < 0.05; red: autism > TD; blue: autism < TD); ×, significant main effect of diagnosis on number of edges (*p* < 0.05; red: autism > TD; blue: autism < TD).


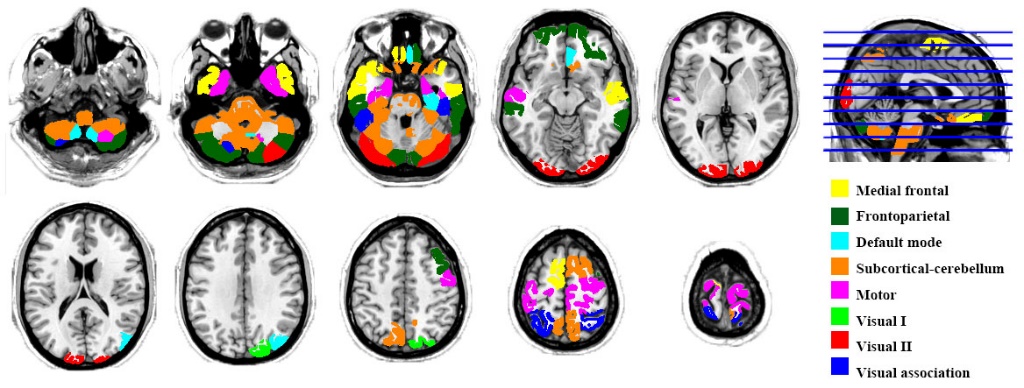


**Figure S4. Regions with incomplete coverage across participants.** Each of eight networks has several regions, primarily located in the parietal lobe and cerebellum, that did not have full coverage in all participants.
